# “When my mind hurts, my body hurts”: Complex PTSD and chronic physical health conditions—A qualitative study exploring the factors contributing to their relationship

**DOI:** 10.1111/bjc.12551

**Published:** 2025-05-27

**Authors:** Laura Blackett, Polly Radcliffe, Teuta Rexhepi‐Johansson, Nicola Reynolds

**Affiliations:** ^1^ Institute of Psychiatry, Psychology and Neuroscience King's College London London UK

**Keywords:** complex PTSD, DSO symptoms, health behaviours, medically unexplained symptoms, physical health, trauma

## Abstract

**Objectives:**

Complex PTSD (cPTSD) has a high comorbidity rate with chronic physical health conditions. This is the first qualitative study to investigate what factors might be contributing to this relationship.

**Methods:**

Twelve participants with cPTSD and chronic physical health conditions were recruited from mental health services across London. Semi‐structured interviews were completed. A reflexive thematic analysis was conducted.

**Results:**

Four themes were identified: Negative Health Behaviours; Mind–Body Link; Negative Core Beliefs about Self, Others and Health; and Negative Impact of Conditions on Wellbeing. The reciprocal relationship between cPTSD and chronic physical health conditions was highlighted: one condition was found to perpetuate or trigger the other, either directly (e.g., pain triggering flashbacks) or indirectly (interfering with treatment). Various factors were found to contribute to the relationship, including cognitive difficulties, sleep difficulties, and reduced social support.

**Conclusions:**

The importance of recognizing mind–body links and targeting factors maintaining both cPTSD and physical health conditions is highlighted.

**Practitioner points**

- Highlights maintaining factors of cPTSD and chronic physical health conditions.
- Makes recommendations for cPTSD treatment targets when there is chronic physical health comorbidity.
- Advances existing research models by highlighting the reciprocal relationship between cPTSD and physical health conditions.
- Identifies the role of Disturbance in Self Organization symptoms which have not been considered by previous models.

## INTRODUCTION

Post Traumatic Stress disorder (PTSD) is a condition that some people develop after experiencing or witnessing a traumatic life event. The International Classification of Diseases (ICD‐11) describes the symptoms of PTSD as re‐experiencing traumatic memories in the present (e.g. experiencing nightmares or flashbacks of the trauma), avoiding reminders of the event(s) (such as thought suppression or not visiting areas with similarities to where the trauma happened) and a sense of threat (increased hypervigilance or beliefs of one as unsafe). Complex Post Traumatic Stress Disorder (cPTSD) was first recognized in the ICD‐11 (Maercker, 2021). It is comprised of the core symptoms of PTSD, as well as Disturbances in Self Organization (DSO): a negative self‐concept (beliefs about oneself as worthlessness or defeated, with pervasive feelings of shame and guilt), difficulties with emotion regulation (heightened emotional outbursts or numbness) and difficulties with relationships (feeling distant from others or struggling to maintain relationships) (Maercker, 2021). CPTSD is often (but not always) a result of multiple traumas that are interpersonal in nature and experienced in childhood (Cloitre et al., 2019; Karatzias et al., 2019).

Compared to PTSD, cPTSD is frequently characterized by higher functional impairment, more comorbidity (Jowett et al., 2020; Ross et al., 2021) and has less favourable outcomes in treatment (Karatzias & Cloitre, 2019). Research has found that women are twice as likely to develop PTSD as men, and symptoms tend to be higher in the younger population and people with unemployment status; however, these findings are inconsistent for individuals with cPTSD (McGinty et al., 2021; Hyland, Murphy, et al., 2017).

Between 50% and 80% of people with PTSD have been found to have chronic physical health symptoms (McAndrew et al., 2019). The physical health conditions include cardiorespiratory disorders, diabetes, medically unexplained symptoms, and functional neurological disorders (FND), as well as gastrointestinal, gynaecological, dermatological, and musculoskeletal disorders (Gray et al., 2020; Iloson et al., 2021; Schnurr, 2022; Schnurr et al., 2000; Seng et al., 2006). The prevalence of chronic physical health conditions has been shown to be higher in people with cPTSD than PTSD (Wright, Roberts, Lewis, et al., 2021).

There are well established biological mechanisms that have been suggested to partly explain the relationship between PTSD and chronic physical health conditions, for example the role of allostatic load (Danese & McEwen, 2012), e.g. chronic stress causing an imbalance in the autonomic nervous system, leading to nervous system inflammation, immune deficit responses, and cardiovascular diseases (Katrinli et al., 2022; Parker et al., 2022).

As well as biological theories, there are four prominent models in the literature highlighting the behavioural, psychological, and physiological connections between PTSD and chronic pain (Gibson, 2012; Otis et al., 2003). For example, the Fear‐Avoidance model (Norton & Asmundson, 2003; Otis et al., 2003) suggests that avoidance plays an important role in the maintenance and development of PTSD and chronic pain. They hypothesise that the avoidance of traumatic reminders (which prevents trauma memories from being processed and maintains an exaggerated perception of threat) or the avoidance of pain stimuli may lead people to isolate themselves and withdraw, which in turn maintains chronic pain and PTSD.

The Shared Vulnerability model (Asmundson & Katz, 2009; Otis & Hardway, 2019) similarly explains the role of avoidance in underlying PTSD and pain but suggests a psychological predisposition underlies this avoidance. The model proposes that anxiety sensitivity predisposes some individuals to become fearful when they experience bodily sensations like pain and after a trauma; this in turn means they are more likely to engage in avoidance behaviours (e.g., withdrawal, low activity) which can lead to the development and maintenance of chronic pain and PTSD within contexts of trauma and somatic symptoms.

The role of anxiety sensitivity and avoidance is incorporated with five other components that are proposed to contribute to both PTSD and chronic pain in The Mutual Maintenance model (Sharp & Harvey, 2001); these are:

1. Anxiety sensitivity (e.g. viewing events as potentially catastrophic)
2. Physical pain as a reminder of the trauma
3. Attentional biases that increase hypervigilance to threat and pain
4. Avoidance coping behaviours
5. Fatigue that drives depression
6. Worries common to both disorders
7. The overwhelming cognitive demands of both conditions limit one's ability to problem solve or use coping skills

This model explains all seven processes that underlie and maintain PTSD and chronic pain, with the cognitive, affective, and behavioural components of pain maintaining and exacerbating the physiological, affective, and behavioural components of PTSD, and vice versa (Asmundson & Katz, 2009).

These three models do not incorporate any of the additional DSO symptoms related to cPTSD. The final prominent model in the literature, highlighting the connections between PTSD and pain, is the only model that inadvertently touches on the role of one of the DSO symptoms (emotion dysregulation). The Triple Vulnerability model (Barlow, 2002; Otis et al., 2003) suggests that for individuals to develop the conditions, they must hold:

1. a biological vulnerability that predisposes them to respond to threats/pain anxiously,
2. a generalized psychological vulnerability from early experiences that instils beliefs of low locus of control and high danger (e.g. “the world is unsafe”),
3. a specific psychological vulnerability where one focuses their anxiety on specific situations (e.g. trauma/pain) and deems these events and their subsequent emotional responses to be unpredictable and uncontrollable.

This model highlights the role of emotional responses (which are more dysregulated in individuals with cPTSD) and of early experiences (childhood trauma is more common in cPTSD).

There are two conceptual models that aim to explain PTSD and poor physical health more generally. Karatzias and Chouliara (2009) propose that PTSD symptoms lead to two types of appraisals: negative appraisals about the body that occurred during the trauma and negative appraisals about the PTSD symptoms themselves. These appraisals subsequently cause negative emotional responses, which increase physiological arousal and negative health behaviours (e.g., substance misuse, avoidance of physical activity), triggering poor physical health. Like the Triple Vulnerability model (Barlow, 2002), it incorporates the role of heightened emotional responses (a common symptom in people with cPTSD) and cognitive beliefs/appraisals. However, the model is more easily applied to individuals who have experienced one trauma rather than multiple traumas, as often seen in individuals with cPTSD.

The second model is described by Schnurr and Green (2004), who proposed that (unless physical injury is caused by the trauma) PTSD is the pathway through which poor physical health develops after trauma exposure, through the following mechanisms:

- Psychological alterations e.g. depression,
- Biological alterations e.g. allostatic load,
- Attentional processes e.g. increased attention towards physical symptoms as a strategy to avoid thinking about the trauma,
- Health risk behaviours e.g. substance use,
- Illness behaviour e.g. reporting of symptoms.

Although there is some evidence supporting each individual factor of the models (Schnurr & Green, 2004), no study has examined the psychological, biological, behavioural, and attentional factors together (Schnurr, 2022). Furthermore, these models do not consider DSO symptoms within their framework.

To our knowledge, only two studies have directly assessed the relationship between DSO symptoms and physical health problems/somatic symptoms (Ho et al., 2021; Kuhar & Zager Kocjan, 2022). Both studies found evidence that DSO symptoms mediated the relationship between childhood trauma and somatic symptoms. Kuhar and Zager Kocjan (2022) found evidence that DSO symptoms indirectly mediated the relationship between childhood trauma and somatic symptoms and that resilient coping (e.g., using strategies to solve problems in stressful situations) decreased the effect of childhood trauma on DSO symptoms. Ho et al. (2021) found that PTSD symptoms and DSO symptoms fully mediated the relationship between childhood trauma and self‐report somatic problems; however, only the PTSD symptoms (and not DSO symptoms) mediated the association between childhood trauma and objective cardiovascular disease load.

It is theorized that DSO symptoms likely contribute to the relationship between cPTSD and chronic physical health conditions. For example, difficulties in managing emotions are likely to contribute to the development of physical health conditions by exacerbating stress levels and triggering biological pathways, such as the allostatic load (Katrinli et al., 2022; Lanius et al., 2010; Parker et al., 2022). This is supported by research finding that emotion regulation mediates the relationship between adverse childhood experiences and physical health (Cloitre et al., 2019), and emotion suppression has been linked with poor physical health (Consedine et al., 2002).

Finally, difficulties with relationships may also contribute to the association. Social support has been highlighted as a protective factor against the development of PTSD (Johansen et al., 2022) and has been associated with better physical and mental health and lower utilization of services in individuals with a history of traumatic experiences (Coker et al., 2003; Lehavot et al., 2013). It is likely that people who experience difficulties with relationships have less social support.

## AIMS

This study aims to address the gap in research by investigating the psychological and behavioural factors contributing to the relationship between cPTSD and chronic physical health conditions, including the role of DSO symptoms. A qualitative exploratory study was chosen over quantitative methods due to its strengths in providing ‘thick descriptions’ to poorly understood events (Willig, 2019). Although quantitative methods have highlighted the links between cPTSD and chronic physical health conditions (e.g. Kuhar & Zager Kocjan, 2022), a qualitative design allowed for a more in‐depth exploration of participants' experiences of living with these conditions and the meaning they made of their experiences.

## METHODS

### Participants

Participants were recruited from primary and secondary mental health services across one NHS London mental health trust. Clinicians at participating services informed service‐users who were eligible for inclusion (e.g., who had a diagnosis of cPTSD and who reported chronic physical health problems) about the study, and participants were able to volunteer for the study using the information provided on recruitment posters advertising the study in waiting rooms. Participants were provided with information about the study, including the study aims, and gained informed consent. Once consented, participants were screened for eligibility to take part by completing the International Trauma Questionnaire (ITQ) and disclosing their physical health condition(s). The ITQ was chosen as it is a valid and reliable measure that assesses cPTSD, in line with the ICD‐11 (Cloitre et al., 2021; Hyland, Shevlin, et al., 2017). Participants were included if they self‐reported a health problem that has been ongoing for at least 6 months, which likely cannot be cured but may be managed through medication or other therapies. It was not a requirement for participants to have a formally diagnosed health condition, as many people with cPTSD report physical or somatic symptoms which may not be found to have a medical explanation and are often undiagnosed (Murray & El‐Leithy, 2022).

Participants were included if they met the requirements for a probable diagnosis of cPTSD according to the ITQ (Cloitre et al., 2018). Participants are required to score ≥ 2 (‘Moderately’) on a 5‐point Likert Scale on at least:

- one of the items relating to each PTSD symptom: (1) re‐experiencing, (2) avoidance, and (3) sense of current threat;
- one display of functional impairment associated with these symptoms;
- one of the items relating to each of the DSO clusters: (1) affective dysregulation, (2) negative self‐concept and (3) disturbances in relationships;
- one display of functional impairment associated with DSO symptoms.

The exclusion criteria included an active diagnosis of psychosis, being 17 years old or younger, and requiring an English interpreter.

### Procedure

The interviewer (Researcher LB) was a white British woman, completing this research as part of a Doctorate in Clinical Psychology, alongside working as a therapist in a secondary mental health service assessing and treating adults with cPTSD. The wider research team consisted of two Clinical Psychologist‐Researchers (with experience treating people with cPTSD and working with mental health needs in physical health settings) and one qualitative Sociologist, Senior Research Fellow. The clinical experience of the interviewer allowed for a fuller assessment of cPTSD beyond the ITQ results. The interviewer helped participants to feel emotionally contained during interviews, enabling them to provide accounts of their subjective understanding and interpretation of the link between their mental and physical health conditions. It should be noted that the research team had more clinical experience in mental health (including working with cPTSD) than they had in physical health. The study was motivated by a particular interest in psychological theory.

The interviewer followed a topic guide (please see supplementary material) which was designed to elicit information about participants' perceptions of the interaction between their physical conditions and cPTSD, including the impact they have on their functioning, beliefs, and coping. Areas of interest were developed from models of PTSD and physical health conditions/chronic pain (Asmundson & Katz, 2009; Barlow, 2002; Karatzias & Chouliara, 2009; Norton & Asmundson, 2003; Otis et al., 2003; Otis & Hardway, 2019; Sharp & Harvey, 2001). Three experts by experience, including individuals living with complex trauma and chronic physical health conditions, reviewed the interview topic guide and protocol, and revisions were made based on their feedback.

The interviews were recorded and transcribed verbatim. Transcripts were checked for errors and anonymised. Participants were reimbursed £20 for their time. All participants were offered the opportunity to make corrections to their transcripts, although no participants did this.

### Data analysis

A thematic analysis was undertaken from a critical realist perspective. Critical realism assumes that a reality exists, independent of one's understanding or knowledge of it (Fleetwood, 2014). Critical realism has been described as combining ontological realism with epistemological relativism (Braun & Clarke, 2022). It was assumed that it would not be possible to observe causal mechanisms and links between physical health and cPTSD directly from the data; instead, interview responses reflect participants' personal and cultural perspectives, interpretations of the interview questions, and demand characteristics. It is the researchers' role to analyse the data to reconstruct their understanding of whether any causal mechanism exists between physical health conditions and cPTSD through the lenses of their own experiences.

A Reflexive Thematic Analysis of the interviews was conducted using Braun and Clarke's (2022) six stage process. A Reflexive Thematic Analysis was chosen as the assumptions embedded within this analysis are appropriate and consistent with a critical realist approach (Braun & Clarke, 2022). Braun and Clarke's reflexive thematic analysis approach is a ‘non‐positivist’ approach, that embraces the subjectivity of researchers as a resource and rejects positivist ideas of researcher bias (Braun & Clarke, 2023). Thematic analysis enables inclusion of participants' subjective understandings and the meanings they give to them, as well as the range of systemic factors that feature in the experience of cPTSD and its relationship to physical health conditions.

Thematic analysis was chosen over Interpretative Phenomenological Analysis because the research question was focused on understanding themes across heterogeneous cases (participants with different physical health conditions and traumas), rather than understanding personal experiences, in a small homogeneous sample size (Braun & Clarke, 2021). Grounded theory was not chosen as the research question was not centred on social processes and instead focused on describing and interpreting patterns in the data and providing a theoretically informed interpretation of themes, rather than developing a grounded theory (Braun & Clarke, 2021).

Researcher LB led on all six phases of the analysis (immersing oneself in the transcripts, coding parts of the data set that were interesting or relevant to the research questions, generating initial themes and sub‐themes, reviewing the themes and defining the themes). All other researchers engaged in inductive coding of two transcripts each, which were compared and collated with Researcher LB's codes, to gain richer insights into the data (Braun & Clarke, 2013). Coding decisions at all phases of the analysis were discussed amongst all researchers until a consensus was agreed.

Once inductive coding was completed, Researcher LB then completed a deductive analysis to capture themes from previous research literature on PTSD and chronic pain/physical health conditions. The benefits of taking a hybrid inductive and deductive approach have been highlighted in research (Neale, 2016) and shown to provide greater rigor in a thematic analysis (Fereday & Muir‐Cochrane, 2006) and to have a strong alignment with a critical realist perspective (Proudfoot, 2023). The deductive coding framework is outlined below.

- Physical pain as a reminder of the trauma (Sharp & Harvey, 2001)
- Avoidant coping behaviours (Asmundson & Katz, 2009; Norton & Asmundson, 2003; Sharp & Harvey, 2001)
- Cognitive demands of both disorders (Sharp & Harvey, 2001)
- Cognitive appraisals (e.g., of vulnerability, low locus of control, high danger) (Barlow, 2002; Karatzias & Chouliara, 2009)
- Negative health behaviours (Karatzias & Chouliara, 2009; Schnurr & Green, 2004)

QSR‐NVivo for Windows (2020) software programme was used to assist with the organization of the data.

## RESULTS

The interviews ranged in length, from 42–79 min, with a mean length of 59 min.

Researcher LB had a prior relationship with one participant prior to the interview due to being her therapist.

### Participant information

Twelve participants took part in the study, four male and eight female. No participants refused to take part or dropped out of the study. Two participants self‐volunteered for the study via recruitment posters, and ten participants were recruited via clinicians within the services. Participants' ages ranged from 23–62 years old, with a mean age of 48 years old. Participants' ethnicity was as follows: White British (*n* = 8), White Irish (*n* = 1), White Other (*n* = 1) and Black Caribbean British (*n* = 2). All participants reported that they spoke English at home, and one participant reported speaking Arabic and English at home.

All participants met the criteria for a probable diagnosis of cPTSD on the ITQ, with a range of scores from 29–47 (sum of the 12 ITQ items) and a mean score of 40.3 (maximum score 48). The interviewer was qualified to diagnose cPTSD and used their clinical experience to ensure participants met the diagnosis of cPTSD. All participants reported prolonged and/or multiple traumatic experiences throughout their life, including child sexual abuse (*n* = 5), other child abuse including emotional, physical, and psychological (*n* = 3), domestic violence (*n* = 4), adult sexual assault (*n* = 3), a car accident (*n* = 1), traumatic events in the workplace (*n* = 1) and time in custody (*n* = 1).

All participants reported at least one chronic physical health condition: two participants reported one condition, two participants reported two conditions, and eight participants reported four or more conditions. Table 1 highlights all the physical health conditions reported.

**TABLE 1:** Self‐reported physical health conditions.

Health conditions	Number of participants with condition
Fibromyalgia	3
Arthritis	4
Carpal tunnel syndrome	3
Diabetes	Type 1: 1; Type 2 Diabetes: 2
Chronic pain	Severe nerve damage: 1; back pain^a^: 3; hip pain: 1; knee pain^a^: 2; neck pain^a^: 1; wide‐spread pain^a^: 1
Asthma	3
High blood pressure	2
Functional neurological disorder	2
Bladder Issues^a^	1
Temporal lobe epilepsy	1
Non‐epileptic attack disorder	1
Poly‐cystic ovaries syndrome	1
Unexplained bowel and abdominal symptoms^a^	1
Cardiac symptoms	1
Long Covid‐19	1
Migraines	1
Stage three kidney failure	1
HIV	1
Thyroid functioning problems	1
Spinal issues	Scoliosis of the Spine: 1; Dislodged Discs in lower spine: 1; Spinal Stenosis: 1

^a^ Undiagnosed conditions.

Four participants reported receiving no welfare benefits, while eight participants reported receiving benefits. Most participants reported not working due to being on long‐term sick (*n* = 2), medically retired (*n* = 1) or unemployed (*n* = 5). Two participants reported working/studying full time and two participants reported working part time. Participants reported a range of education levels from less than secondary school (*n* = 2) to PHD (*n* = 1).

### Themes

**FIGURE 1:**
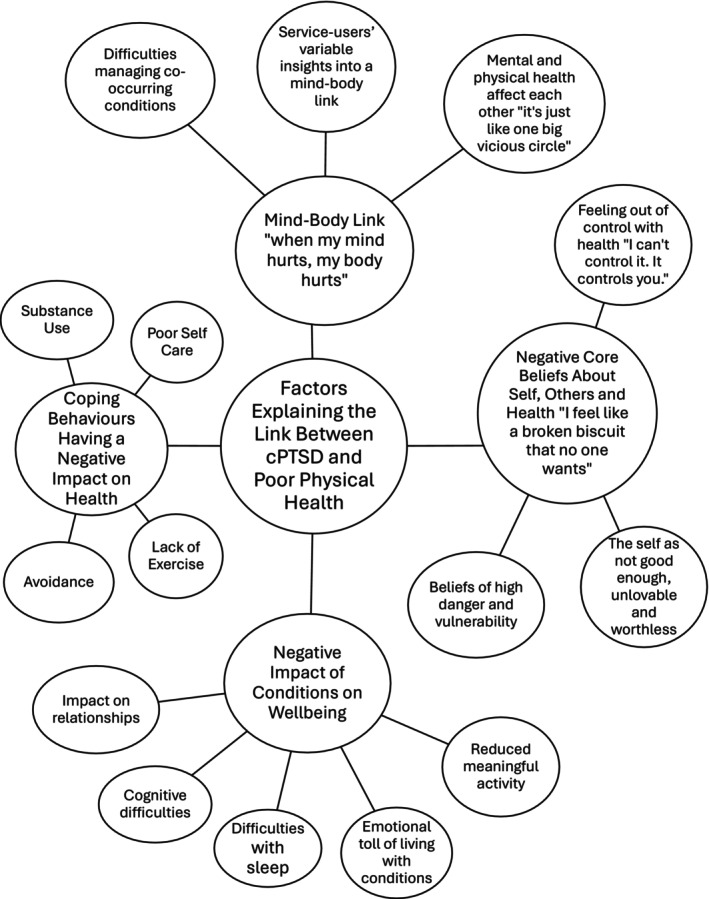
Thematic map of factors explaining the link between cPTSD and chronic physical health conditions.

Four themes were identified: (1) Coping Behaviours Having a Negative Impact on Health; (2) Negative Impact of Condition on Wellbeing; (3) Mind–Body Link “When my mind hurts, my body hurts”; (4) Negative Core Beliefs About Self, Others and Health “I feel like a broken biscuit that no one wants”. The themes and sub‐themes are presented visually in Figure 1. The frequency of codes in each theme and sub‐theme are shown in the supplementary materials.

#### Theme 1: Coping behaviours having a negative impact on health

A range of behaviours that have a negative impact on health (negative health behaviours) was reported by participants in response to cPTSD and related chronic physical health conditions, including substance use; poor self‐care; lack of exercise; and avoidance. Often, participants reported that these behaviours were an attempt to manage one condition (e.g., using alcohol to manage cPTSD symptoms) but had secondary negative consequences for their other conditions (e.g., worsening physical health symptoms).

##### Substance use

Participants reported using substances, including tobacco, alcohol and illicit and prescribed drugs, to help ‘escape’ from their flashbacks and nightmares, for pain relief, to manage stress, to regulate emotions and as a form of self‐harm.

I got an addiction of smoking because it's so stressful. P3

Some participants recognized the impact of substance use on their physical health conditions, but nonetheless continued to use substances.

Interviewer: “Is there anything you do to manage your physical and mental health symptoms?” Participant “I smoke rollups”…“I shouldn't, I know I shouldn't because it's not good for diabetes. It doesn't make any sense.” P2

Participants reported prescribed medication non‐adherence and side‐effects, for example taking more physical health medication than prescribed to manage flashbacks and to help them sleep. Other participants reported underusing their prescribed medication, for fear of becoming addicted or feelings that they were already taking too much medication.

I've taken them closer than you're meant to, when you're meant to take them 12 hourly I've taken them, you know, four hourly or whatever…I think it just helps me to get to a point where I'm so tired that I'm definitely gonna go to sleep and I'm not gonna be woken by a memory or whatever. P5

##### Poor self‐care

Some participants reported that ‘self‐destructive feelings’ and low mood led to a failure to look after themselves properly, including poor hygiene, poor diet, over‐work or failing to care for injuries.

It's very destructive my mind. I don't eat properly and I don't care about anything. Excuse my language, F this, you know, All that sort of stuff. P8

##### Lack of exercise

Participants reported not engaging in physical activity due to their physical health symptoms (e.g. pain), their low mood, or poor motivation. Most participants recalled negative effects on their physical and mental health due to not exercising.

I think in dealing with my mental health, exercise is very important, and, you know, I feel so much better when I exercise, but I feel it's restricted because of my asthma. P6

I do feel a bit better when I do the little bit exercises, but it's just getting to do them, which isn't a very often. P1

##### Avoidance

A range of avoidant coping behaviours was highlighted by participants, including withdrawing, looking for distractions from their problems, avoiding thinking/talking about their conditions and trauma, and avoiding health care. Participants reported avoiding seeking health care for fear that their physical health would be dismissed or not believed (often related to previous negative experiences of health care), ignoring the need for treatment (sometimes until a crisis point) and not wanting to waste services' time.

“First time I got into the doctors in a long‐time ‘cause the doctors is my scared place because yous lot didn't listen”….“I didn't know what PTSD was, I just thought it was normal, you know having flashbacks of a past that you never wanted to see again.” P3

Many individuals reported hiding their trauma and physical health conditions from health care professionals, their employer and friends and family, due to shame, fear of not being believed and assuming others will not understand their conditions. Hiding conditions decreased opportunities for finding support and often participants reported that their conditions declined further.

I found it hard to talk about why, or to say this is going on, because you get, you're so embarrassed, and so ashamed, and so used to not being heard, and not being taken seriously, or just called an outright liar, that you don't volunteer information. So, I can see that, to some degree, it could be my responsibility as well. P7

Some participants reported that once they had admitted their trauma histories to professionals, they finally received the right treatment.

It was when I left [abusive partner] maybe 2015/16. That's when I got to the correct people. I mean, they knew I was having seizures they just didn't know the actual reason why, who done it. P4

#### Theme 2. Negative impact of conditions on wellbeing

Living with cPTSD and chronic physical health conditions was observed to have a widespread impact on participants' wellbeing. Five sub‐themes were identified: (1) Reduced meaningful activity, (2) Emotional toll of living with conditions, (3) Difficulties with sleep, (4) Cognitive difficulties, and (5) Impact on relationships.

##### Reduced meaningful activity

All participants reported that their chronic physical health conditions and cPTSD symptoms prevented them from carrying out everyday tasks and meaningful activities. For example, they reported that their pain, fatigue, low mood and/or PTSD symptoms interfered with their ability to engage in social, religious, and family activities, as well as fitness and leisure hobbies.

It [fibromyalgia] stopped me going to church. It stopped me looking after my grandchild. It's stopped me going to my mental health group. It's stopped me, leading a Bible study group that I'm meant to do every week. P7

Participants also reported that the conditions impacted their work or education, which had financial consequences and generally limited their relationships and quality of life.

This whole time I haven't been working because I've got no control, and no one will hire me if the seizures aren't controlled. P4

##### Emotional toll of living with conditions

All participants reported an emotional toll of living with cPTSD and chronic physical health conditions, including expressing high levels of distress, guilt, anger, anxiety, and low mood. Some participants reported feeling overwhelmed and unable to cope and had attempted suicide or had suicidal thoughts.

“But the pain then travels. So, I'm in a no‐win situation when it comes to that. So yeah, I get really anxious with myself. I get so angry”…“last year February I took an overdose, wrote my son a letter cause life was just done. I physically I can deal with anxiety, and I can deal with depression, but I cannot deal with my PTSD.” P3

Many participants reported shock and distress in relation to being diagnosed with their chronic physical health condition, with some people suggesting the physical health diagnosis was ‘another trauma’.

I had type one diabetes and I think that in itself can be quite traumatic to just, and at my age as well that I was, I was 17. P2

##### Difficulties with sleep

Participants reported difficulties sleeping because of both their cPTSD symptoms and physical pain. The poor sleep was reported to perpetuate physical and mental health symptoms.

If I go to sleep and I'm in a bad mood, like feeling crap then my mind is in overdrive. But also, I'm also fighting going to bed on my back, I'm getting the shooting pains in my legs and that. It's just like double whammy. P11

When I'm sleep deprived, that triggers seizures. P4

##### Cognitive difficulties

During the interviews, participants described and exhibited cognitive difficulties which were related to PTSD symptoms, pain, physical health medication and FND symptoms. This affected their view of themselves and their overall wellbeing.

Kind of like an intellectual toll on, how do I put it, operating a body that hurts every time you move it, that takes a lot out of your kind of, like, say, emotional and mental reservoir. P12

The management of physical health conditions was also described as cognitively demanding, and cognitive difficulties were reported to interfere with treatment of both physical and mental health conditions.

The dose adjustment, learning how much to take and things like sick day rules, because, you know, when you're ill, you may have to take more insulin. So it's quite, it's quite tricky.

Some participants described only being able to attend to one condition at a time, which affected the management of the conditions.

Yeah, it's just like dealing with two hot stones and your brain can't focus on either of them and then I'll just get to the point. Not going to do nothing then. P11

##### Impact on relationships

Participants reported many ways in which their physical and mental health difficulties impacted on their relationships. For example, irritability and anger (related to PTSD symptoms or anxiety around managing physical health) were reported to cause conflict in relationships. Pain, fatigue, or cPTSD symptoms meant they did not want to engage with other people or they lost contact with friends due to the losses they experienced related to their physical health conditions.

If my sugar level is low and I'm feeling shaky and I'm getting a bit arsey and I'm like, “Just get me a drink, man!”. P2

Participants reported that they require more care in their relationships due to their conditions. Some participants reported feeling worried about their reliance on others or feeling guilty for being the receiver of support.

Sometimes I just think to myself, ‘How would I cope if he wasn't around’? You know, so I don't want to take advantage of those that I need. P2

Other people reported that they do not ask or accept help from others for fear of burdening them, feeling embarrassed or expecting others to be unhelpful due to strained family relationships.

“I've had it from family as well. “It's like having a child again”. So that's why I don't ask for help, because I'm embarrassed by that. I don't want to be seen as a child”…“if I don't have much support from the people closest to me, how will anyone believe me, if that makes sense?” P4

Some participants reported that the little social support available to them has had a negative impact on them.

So that kind of doesn't help because I haven't got a circle of people that I can really count on. P1

#### Theme 3. Mind–body link “When my mind hurts, my body hurts”

This theme refers to participants' perceptions of how their cPTSD symptoms (mind) and their physical health symptoms (body) interacted with one another, often with one condition perpetuating the other. Three subthemes were identified: (1) Mental and physical health affect each other “it's just like one big vicious circle”; (2) Difficulties managing co‐occurring conditions; and (3) Service‐users' variable insights into a mind–body link.

##### Mental and physical health affect each other “it's just like one big vicious circle”

Physical health symptoms, such as pain and seizures, were reported to impact on mental health, such as causing emotional distress, lowering mood, and triggering flashbacks and dissociation (where one disconnects from their body and/or the world around them). In some cases, pain functioned as a reminder of the trauma experienced, either through mirroring the physical sensations or the feeling of being in ‘danger’.

[Developing epilepsy] triggered everything for me, like from my past. Obviously, it was always there, and I have flashbacks from what had happened to me, but it sort of made it more intense. P10

Sometimes at night, when my neck is hurting or I move, that will trigger a flashback of being held, pinned down. P7

Mental health was also found to directly affect physical health symptoms. Participants reported that cPTSD symptoms and low mood exacerbated physical health symptoms and pain.

So, the flashbacks affect my mood, which then affects my pain. P1

Moreover, participants reported that their mental and physical health symptoms would interact in a vicious cycle and increase overall suffering.

I couldn't control the seizures and they were getting worse and as my mental health was obviously getting worse, the seizures were getting worse. P4

Three participants recognized that they needed to look after their mental health to look after their physical health.

It will be a like a circle, because if I don't look after myself, I'm just going to be sick and so mentally, I have to make sure that I'm okay in order to look after myself. P2

##### Difficulties managing co‐occurring conditions

Participants reported difficulties managing co‐occurring conditions, due to each condition having opposing needs or interfering with the management of the other. For example, physical health conditions sometimes interfered with or negatively affected mental health treatment or activities that were helpful for one's mental health, and vice versa.

I didn't want my[self] [physical health condition] to get better. My mental health was bad, so in like almost a self‐harm kind of way, [I chose] to not seek medical help. P6

In some cases, the treatment for one condition was reported to have a direct negative impact on other conditions.

I've had lots of seizures going through all this treatment with [therapist name]. You know, like bringing up my past and yeah, I'm emotionally drained. I've seizured, but she did say that could happen. P10

##### Service‐users' variable insights into a mind–body link

When asked about whether they believe there is a link between their physical and mental health, service‐users gave variable responses. Half of participants reported a clear link between their physical and mental health at some point during the interview.

If my mental health goes down, my physical health goes down, it's linked. My body can't cope—when my head can't cope, my body can't cope. P4

At other times during the interview, participants did not report a link, however, discrepancies were observed in their statements later in the interview. Some participants reported a change in their understanding of the link between their cPTSD and physical symptoms. One participant noted that engaging in the interview had improved their understanding. One participant recognized that they had to be ready to see the link for it to be a helpful suggestion from professionals.

It probably would have been a nonstarter because I wouldn't have made that link. I wasn't at that stage yet. P12

#### Theme 4. Negative Core beliefs about self, others and health “I feel like a broken biscuit that no one wants”

This theme recognizes the role which core beliefs play in the development and maintenance of cPTSD and chronic physical health conditions. Three sub‐themes were identified: (1) feeling out of control with health “I can't control it. It controls you.”; (2) The self as not good enough, unlovable and worthless; and (3) Beliefs of high danger and vulnerability.

##### Feeling out of control with health “I can't control it. It controls you”

This sub‐theme focuses on participants' beliefs about the controllability of their cPTSD and physical health conditions.

Other than diabetes and asthma, which were reported to be well controlled by medication by three participants, a low locus of control of mental and physical health conditions was reported. Low perceived control of physical health symptoms, PTSD symptoms, and emotion regulation were linked to feeling scared and contributed to negative core beliefs of self as vulnerable, which increased avoidance of socializing with others. There were varying views as to whether techniques and treatment helped increase control.

I can't control it. It [cPTSD] controls you. P8

I think therapy helps to control it, but you know, I think things are suggested to help it and then, you know, rely on that to help it, but it doesn't. P6

Most participants hoped that they would be able to manage their conditions better in the future. However, some participants reported feeling hopeless and fearing that their conditions were incurable or would decline with time.

But I think I hold out hope that maybe there will be a cure for it, but that's not grounded in reality. It's just some hope I've got. P8

##### The self as not good enough, unlovable and worthless

All participants reported negative beliefs about themselves and about how others view them. For example, participants reported feeling not good enough, abnormal, like a failure, mad/crazy, and worthless.

I feel a bit like a broken biscuit. You know, like the broken biscuit in the bottom of the jar that no one wants. P11

it just makes me feel like I haven't done anything good? Like, I'm not good enough. Let myself down. P1

Furthermore, experiences of not having their physical health symptoms believed by health care professionals, or not having an explanation for them, perpetuated self‐blame and negative core beliefs.

“I felt I blamed myself because why can't I be stronger? If it's not that serious, I took it “well it's not that bad then is it?”, “it's just your head.” P4

##### Beliefs of high danger and vulnerability

Participants reported increased feelings of vulnerability and weakness linked to traumatic experiences, physical health symptoms, and physical disabilities.

“I get anxious towards people, I'll give myself that extra distance to get away” … “I'm not so mobile, so I need that extra time to get away” P11

Their responses also suggested they held beliefs that others are dangerous, untrustworthy, and judgemental.

Nowadays, people will rob you and hurt you, they wouldn't help you. P10

### Visual diagram of connecting factors

**FIGURE 2:**
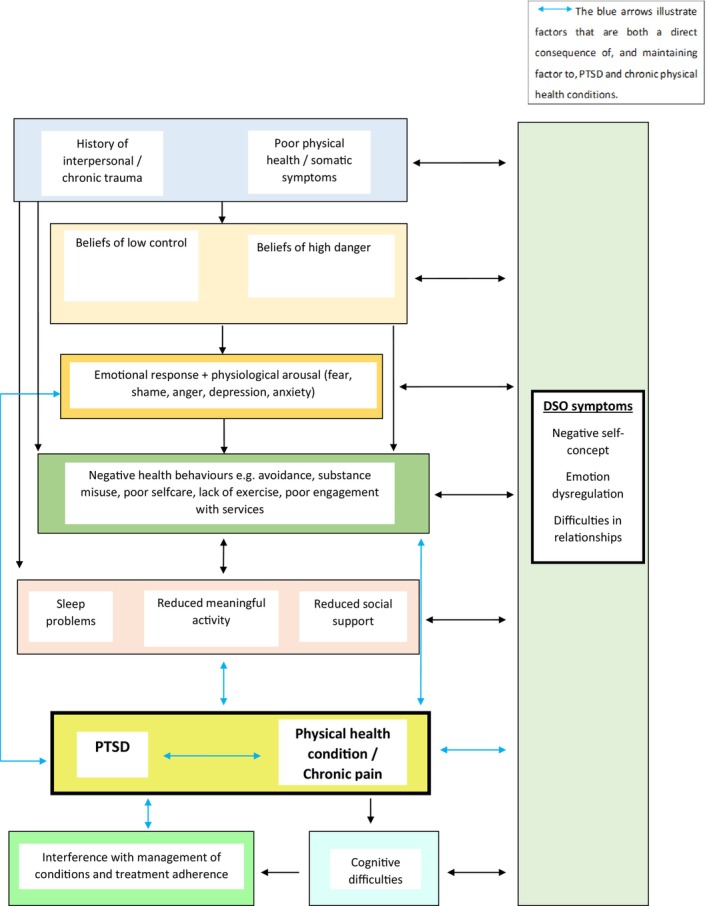
Factors contributing to cPTSD and chronic physical health conditions.

Figure 2 provides a visual representation of how factors were found to connect to each other and to participants' cPTSD and chronic physical health conditions. This figure is not used to imply causality. An example of a pathway between the different factors is found below.

All participants reported a history of chronic trauma and physical health symptoms. Most participants described low perceived control over their symptoms (“*I can't control it. It controls you*”), along with a sense of high danger (“*people will rob you and hurt you, they won't help you*”), which was linked to strong emotional responses (e.g. feeling scared/vulnerable) and avoidance of relationships, exacerbated by poor emotion regulation (DSO symptom). As a way of attempting to cope with their distress, shame/guilt (negative self‐concept) and symptoms of their conditions, they reported engaging in negative health behaviours, such as substance misuse and avoidance (“*why I don't ask for help, because I'm embarrassed*”). Often as a consequence of this, participants complained of sleep problems, reduced meaningful activity and reduced social support, along with reporting they contributed to both PTSD and physical health conditions and DSO symptoms (e.g. avoidance reinforced difficulties in relationships). Poor memory and attention were also reported because of the conditions and linked to forgetting appointments, medication and contributing to negative view of self (e.g. “I'm stupid”).

## DISCUSSION

The findings of this study advance existing evidence regarding the nature of the relationship between PTSD and chronic physical health conditions. Although further research is needed, a number of preliminary psychological, cognitive, and behavioural factors have been found to have a bi‐directional relationship with cPTSD, highlighting the importance of DSO symptoms.

As recognized in the Mutual Maintenance model of chronic pain and PTSD (Sharp & Harvey, 2001), this study supports the idea that pain (and other physical health conditions, such as seizures) acts as a reminder of the trauma (either through mirroring physical sensations or feelings of being in danger) and could trigger flashbacks and increase distress. Furthermore, the Mutual Maintenance model's (Sharp & Harvey, 2001) proposal that the cognitive demands of both conditions together limit an individual's ability to problem solve or to use coping skills was also supported by our findings, with participants reporting they only had the cognitive capacity to attend to the management of one condition at a time (i.e. either cPTSD or physical symptoms).

In line with the Triple Vulnerability model (Barlow, 2002), appraisals of low control (of symptoms and subsequent emotional responses) and high danger were found to be important in the development of cPTSD and chronic physical health conditions. Appraisals of ‘high danger’, such as ‘I am vulnerable and weak’ and ‘others are dangerous’, were reported as a response to traumatic experiences and their physical health conditions (e.g. due to poor mobility or complex health needs). These appraisals interacted with individuals' engagement in negative health behaviours (particularly avoidance), along with impacting on their relationships (e.g. caution of others), negative self‐concept (e.g. feeling not good enough/shame), and emotional dysregulation.

Appraisals of low control over conditions were linked to feeling scared, helpless, and vulnerable and interacted with individual's engagement with treatment (e.g. feeling reliant on treatment for control or not engaging due to perceiving treatment as unhelpful for ‘uncontrollable’ conditions). Furthermore, this contributed to participants avoiding meaningful activities and socializing (until they felt more ‘in control’ of conditions), which likely impacted on their relationships and mood.

Moreover, the findings of this study are also consistent with research that reports the negative implications of poor sleep on physical health and the development of psychiatric disorders, including PTSD (Freeman et al., 2020; Hanson & Huecker, 2019; Karatzias & Chouliar, 2009; Schnurr & Green, 2004; Sharp & Harvey, 2001; Tang, 2009), suggesting that sleep problems may be a bridge between PTSD and somatic symptoms (Wright, Roberts, Barawi, et al., 2021).

Other parts of Karatzias and Chouliar (2009) and Schnurr and Green (2004)'s research also supported. High levels of distress (emotionality) were found to contribute to the relationship between cPTSD and physical health, by leading to negative health behaviours, including poor self‐care, a lack of exercise, substance misuse and avoidant coping. This also supports pain and PTSD models that have incorporated avoidance as a mechanism underlying the conditions (Asmundson & Katz, 2009; Norton & Asmundson, 2003; Sharp & Harvey, 2001). The findings of this study highlighted that physical health conditions may also be driving these behaviours and contributing to the development of both conditions. For example, asthma and pain were reported as reasons for being unable to engage in exercise and seizures were reported to trigger emotion dysregulation and PTSD symptoms (e.g. flashbacks).

The bidirectionality of the relationship between cPTSD and chronic physical health conditions is an important finding and was particularly highlighted within the theme ‘mind–body link’. CPTSD symptoms and chronic physical health conditions were described to interact with one another in a vicious cycle, perpetuating symptoms of the other condition both directly (e.g. “when my mind hurts, my body hurts”) and indirectly (e.g., conditions were sometimes observed to have opposing needs and one condition often interfered with the management of the other).

### DSO symptoms

DSO symptoms were identified to have a bidirectional relationship with both PTSD and chronic physical health conditions, as well as with other maintaining factors.

### Difficulties with relationships

Difficulties with relationships were shown to be linked to both PTSD and physical health conditions: symptoms lead to conflict within relationships and avoidance of relationships. This can create a vicious cycle; for example, individuals experience more difficulties in relationships due to their conditions (e.g. needing extra support / adjustments), they are also likely to struggle to navigate these difficulties with others due to their interpersonal difficulties or beliefs that others are judgmental or dangerous (likely derived from early developmental or interpersonal trauma).

### Emotion dysregulation

The emotional consequences of living with cPTSD and chronic physical health problems, often due to difficulties in adjusting to the disorders, have been documented in this study, in line with previous research (Ehring & Quack, 2010; Herrera‐Escobar et al., 2021; Kinsella & Moya, 2021).

High levels of distress were shown to contribute to participants' cognitive difficulties (e.g. increased dissociation), engagement in negative health behaviours, sleep problems, and reduced social support, all of which were reported to contribute to the development and maintenance of both disorders. Difficulties regulating emotions (a symptom of cPTSD) likely exacerbates emotional distress and impact on the maintenance/development of the conditions.

Emotion dysregulation also contributes to the development of physical health conditions by triggering biological pathways, such as the allostatic load (Katrinli et al., 2022; Lanius et al., 2010; Parker et al., 2022), suggesting individuals with cPTSD (and poor emotion regulation) may be at a higher risk of developing physical health conditions.

### Negative self‐concept

A negative self‐concept was found to be both a consequence of cPTSD and physical health conditions and a contributor to maintaining the conditions.

A negative self‐concept interfered with individuals seeking treatment or support from services or friends/family, contributing to a deterioration in conditions. For example, some participants reported that they did not initially ask for help due to not wanting to waste services' time (e.g. feeling unworthy of support) or not wanting to tell professionals about their traumatic experiences or cPTSD symptoms due to feelings of shame, self‐blame, and feeling like a failure.

This study also identified that living with additional chronic physical health conditions maintained negative beliefs and impacted on cPTSD symptoms. For example, physical health symptoms and associated disability were found to increase feelings of vulnerability and danger (contributing to PTSD symptoms) and reinforcing beliefs of being weak or not good enough.

Research has also found strong links between physical illness and low self‐esteem and high levels of guilt (Cerna et al., 2022; DeLongis et al., 1988; Glendinning & Inglis, 1999), in line with this study where heightened levels of guilt were reported for often being the receiver of support in relationships due to their health needs.

### Clinical implications

This study highlights a need for further research into the interaction between chronic physical health conditions and cPTSD. Preliminary findings from this study are in line with current agendas (Das et al., 2016; NHS England, 2016; Spilsbury, 2017) which suggest a move towards more integrated physical and mental healthcare for better outcomes.

New holistic, multi‐disciplinary treatment programmes which target chronic physical health conditions and cPTSD together should be tested, including the development and clinical trial of trauma‐focused therapies which also target DSO symptoms, physical health conditions, sleep difficulties, cognitive appraisals, and negative health behaviours, rather than PTSD symptoms alone.

Although further research is needed due to the limitations of this study, preliminary findings suggesttherapists attention should be paid to the emotional impact of physical symptoms, to whether physical symptoms mirror traumatic experiences (and, if so, whether this triggers PTSD symptoms) and to any maintaining cycles between cPTSD and physical health conditions.

Service‐users and research participants may need support in overcoming barriers to engagement, including the likely increased cognitive demands which both conditions place on them, and researchers or services should provide reminders for appointments and more visual and written information.

### Limitations

A number of limitations should be considered when generalizing the results. The study consisted of a small sample (*n* = 12) of mainly white (*n* = 10) and female (*n* = 8) participants. It cannot be assumed that similar themes would have arisen in a more diverse sample, particularly as research has found gender and ethnicity differences in experiences of physical health (Conversano et al., 2021) and cPTSD symptoms (Street & Dardis, 2018; Weiss et al., 2022).

The researchers' expertise was based in clinical psychology, and the deductive framework used within the research was developed from models using psychological theory to link PTSD with chronic physical health conditions/chronic pain, thus less attention may have been paid to biological explanations or physical health themes. For one participant included in our study, their traumatic experience was the cause of their physical health condition, which may result in different cognitive appraisals about their condition. This should be considered further in future studies.

Furthermore, participants (with cPTSD diagnoses) were recruited from mental health services, so the results may differ from people with cPTSD and physical health conditions who have only accessed physical health services or who have not sought help for their conditions. Future research should aim to explore the relationship between cPTSD and chronic physical health conditions in patients recruited from physical health services, such as GP practices, chronic pain clinics, and A&E. Investigating the perceptions of medical professionals, such as GPs, and how they manage patients with these symptoms should also be considered.

Finally, as the study relied solely on participant responses, factors outside of participants' awareness may have been missed. Attentional biases, which have been suggested to underlie PTSD and chronic pain/physical health conditions in previous research (Barlow, 2002; Schnurr & Green, 2004; Sharp & Harvey, 2001), could not be identified in this study design but may still be an important factor to consider.

## CONCLUSIONS

This study highlights a range of psychological, behavioural, and cognitive factors that contributed to the relationship between cPTSD and chronic physical health conditions. Some factors have been highlighted previously by proposed models of PTSD and chronic pain/physical health conditions, such as the role of cognitive appraisals (particularly of low control and high danger), poor sleep, cognitive difficulties, and negative health behaviours (e.g. substance misuse and avoidance). However, this study identifies reduced social support and DSO symptoms, which have not been considered by previous models. This study advances existing research by finding that these factors have a bidirectional relationship with cPTSD and chronic physical health conditions and highlights the reciprocal relationship between the two. One condition was found to perpetuate or trigger symptoms of the other, either directly (such as with physical health symptoms triggering flashbacks) or indirectly (by interfering with management and treatment). The importance of recognizing the links between the mind and body and the need for further research into the interaction between chronic physical health conditions and cPTSD was highlighted. New holistic treatment programmes that target chronic physical health conditions and cPTSD together should be trialled.

## AUTHOR CONTRIBUTIONS

**Laura Blackett:** Conceptualization; investigation; writing – original draft; methodology; formal analysis; project administration. **Polly Radcliffe:** Conceptualization; writing – review and editing; methodology; formal analysis; supervision. **Teuta Rexhepi‐Johansson:** Writing – review and editing; conceptualization; methodology; supervision; formal analysis. **Nicola Reynolds:** Conceptualization; methodology; writing – review and editing; supervision; formal analysis.

## CONFLICT OF INTEREST STATEMENT

The author(s) declare that there were no conflicts of interest with respect to the authorship or the publication of this article.

## ETHICAL APPROVAL

This study received NHS ethical approval from the Northwest Greater Manchester Research Ethics Committee (IRAS reference: 22/NW/0193). Research governance approval to recruit from adult mental health services was granted by the Joint Research and Development Office of South London and Maudsley NHS Foundation Trust and the Institute of Psychiatry, Psychology and Neuroscience (Trust R&D Ref: R&D2022/060).

## Supporting information


**Data S1.** Supporting Information.

## Data Availability

The data that support the findings of this study are unavailable due to privacy or ethical restrictions.
