# Supplementary material for: “When my mind hurts, my body hurts”: Complex PTSD and chronic physical health conditions—A qualitative study exploring the factors contributing to their relationship
Source: Br J Clin Psychol. 2025 May 27;64(4):1020–42. doi: 10.1111/bjc.12551 (PMC12506954; doi:10.1111/bjc.12551)
Supplement: Supplementary file 1 — Data S1. Supporting Information. [file BJC-64-1020-s001.docx]

**
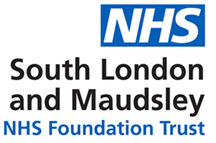
9. Supplementary Material**

**Interview Topic Guide**

Current physical health:

- Please can you tell me a bit about your physical health condition(s) and, if any, your current treatment for this?

**Prompt**: How long have you had these difficulties? When did you get diagnosed? What health care professionals and services you have been in contact with about your physical health condition?

Care Pathway:

- We understand that accessing services is not always straight forward. What health care professionals and services have you been in contact with, in relation to your complex PTSD?

**Prompt:** When did you first seek help? How? Did anyone else seek help for you? Did you first seek help for your complex PTSD or your [inset physical health condition]?

- What diagnoses came first? Are there any other health care professionals and services you have been in contact with?

**Prompt:** Has anything been helpful about your journey through services to [insert current service] so far?

**Prompt:** Would anything have improved your journey to [insert current service]? What? Did professionals communicate the way you would like them to? Have professionals taken the time to learn about you?

**Prompt:** Has Covid-19 affected your care?

The Relationship Between Physical and Mental Health

- Do you think there is a link between your physical and mental health? Please describe further.

**Prompt**: Do you notice a change in your mental health, when your [physical health condition/pain] changes? What about when your mental / physical health improves / is triggered [e.g. you experience flashbacks, emotional distress, feeling stressed / anxious / angry]? Is there anything that leads your physical / mental health to get worse? Does your physical health affect you emotionally? If so, how?

**Prompt:** Have you noticed any effects on your physical health due to your mental health treatment? Have you noticed any effects on your mental health due to your physical health treatment?

**Prompt**: Has your mental health and physical health difficulties been treated separately, or were both taken into consideration in your appointments with mental and physical services? What would be your preference?

Information Guide. IRAS number: 30968. Version 1.0. Date: 24.03.22

Cognitive appraisals


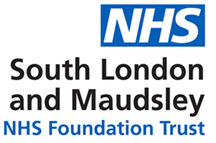


- Does your physical and mental health impact the way you view yourself? How?

**Prompt:** How do you think others view you / your physical health / mental health?

- What do you think has caused your physical health problems?
- Do you think you can control your physical health problems?

**Prompt:** How? To what degree? Do you see your health changing in the future? How?

Do you think your physical health problem can be treated? To what degree?

- What about your complex PTSD: can you control this?

**Prompt:** How? To what degree? Do you see your cPTSD changing in the future? How? Do you think your cPTSD can be treated? To what degree?

Coping Strategies and Health-Related Behaviours

- Is there anything you can do to help manage your physical / mental health symptoms?

**Prompt:** What support do you have for your physical / mental health? What has been most helpful? What has been less helpful? Has anything made things worse?

We know it is common for people to use drugs and alcohol to help cope. Do you currently, or have you previously taken drugs or medication to help manage your physical / mental health? What did /do you take / what dose/ how often? Have you ever taken more than the prescribed amount? Do you currently, or have you previously used alcohol or any other substances to help manage your physical / mental health? What? Have you ever received treatment for drug or alcohol use?

Functioning and Impact

- Has your physical / mental health affected your life?

**Prompt:** If you didn’t have physical / mental health difficulties, what would be different?

Has it affected your relationships? Career? Hobbies? How?

Does your physical health affect your ability to engage in your mental health treatment? Does your mental health affect your ability to engage in your physical health treatment?

Debrief

- Offer grounding / relaxation techniques
- Offer chance to learn about results of study.
- Offer check-in in 3 days’ time.
- Offer to send transcripts of interview to them once collected.
- Discuss £20 reimbursement.

Additional Quotes as evidence of themes:

Information Guide. IRAS number: 30968. Version 1.0. Date: 24.03.22

Information Guide. IRAS number: 30968. Version 1.0. Date: 24.03.22

Theme 1: Coping Behaviours Having a Negative Impact on Health

*Substance use*

*“as a form of self-harm with the alcohol or just wanting to feel, not feel anything, numbness, and the drugs was, that was cocaine so that was quite bad, and that was wanting to also escape and make myself feel better, and obviously cocaine that’s such an addictive drug.” P6*

*“I could erm come home from stressful things that I've seen at work and dealt with and that was the way you dealt with it. I mean, I could come in on off a night where it's been particularly bad and I could spin the lid off a bottle of Scotch and do a bottle of Scotch before going to bed.” P5*

*“I don't take pain meds just because I had them for so many years, to the point that was just damaging my body.” P3*

*“I’m on an antidepressant and an antipsychotic and I’m taking more of my antipsychotic and my GP said don’t do that.” P8*

*Poor self-care*

*“I've been so low this week. I've cried every day”…”I forget, I haven't showered for three days, so yeah, I haven't washed my hair for, like, a week and a half.”… “I was so overworked, overstressed… my Dad said all you did was work, work, work… you didn’t have you time.” P10*

*Lack of exercise*

*“I just want the pain to stop so that I can get on with my life, get out on my bike, cycle around, have a good cycle around, I used to belong to a cycle group, that is a mental health group.” P9*

*Avoidance*

*“like someone just said, ‘you need to see someone, cos you're like a ticking time bomb’. And then I basically just tried to ignore it [mental health struggles] and just pack it* away. And time *catches up on you, doesn't it?” P11*

*“I cannot deal with my PTSD. I locked myself in my bedroom, which I've done from as young as seven years old. So it's like a thing that's literally followed me along, and there's no way out”… “I’d take on people's problems and forget my own, but then come back home and struggle” P3*

*“I shut myself away for about four years because I didn't, I couldn't explain it. I didn't understand it.” P4*

*“I mean on the pain front I've just got, I've just literally when it gets really bad, I literally go to bed.” P5*

*“My Mum was like, ‘Oh, let’s get an ambulance’. I was like ‘No, let’s not waste their time’.” P2*

*“I’ve been struggling for about 12 years, but I had a breakdown and couldn’t work, then I started seeing my GP. Before I was just coping at home, you know, really bad.” P8*

*“I've had flashbacks for those which obviously I've never ever bothered seek treatment or anything like that” P5*

*“I wait a long time before I’m in the company to disclose that [mental health], same with my physical health.”…“I was here for say 3 ½ years before I opened up about mental health because I literally worked to cover it up.” P3*

*“There is absolutely no way in the world I would have disclosed anything [trauma] whatsoever because to me that would feel like failure. Yeah, because I’ve failed in that, everybody else can cope with it so why can't I?” P5*

*“I don’t like anyone seeing me as vulnerable, I wanted it to be my choice if I tell someone” P4*

Theme 2. Negative Impact of Conditions on Wellbeing

*Reduced meaningful activity*

*“there's so much pain and every day is just a constant battle to do the simplest things. And I just feel so exhausted from trying to do those things that then some higher functioning things like paying bills and answering emails like I, you know, made it to and from work, what more does the world want from me like today?” P12*

*“It probably might help me if I get up and go out, but it's just I can't. I just haven't got the energy to get ready. I haven't got the energy to even think about combing my hair because it hurts when I lift my arms like, yeah. So I thought, why put myself through all of that? I just stay in the house.” P1*

*“If I didn’t have epilepsy, I’d have no worries. If I didn’t have flashbacks, I’d be able to do so much, I’d be able to take the kids out again like on the weekends.” P10*

*“I'll take my insulin so everything was more controlled. My mental health seemed fine, but I have noticed now how much it affects, it affects my ability to do stuff. So therefore, it will affect my mental health because you know, where some others can go and do all this stuff and that they've got energy, me, l can't.” P2*

“*I'm not married. I mean, I don't have a career, so I'm living on benefits money, which is very little. Can't do anything. I try to do things; I can get triggered really easily and I have to have time out. Yeah. So it's very limiting.” P8*

*Emotional toll of living with conditions*

*“I get very angry, like if something like touches me. So, like, a bit of water splashes on, I feel like smashing things up.” P8*

*“Psychologically, it was quite a hard one to be diagnosed with because my mum has type two diabetes and has for a long time, and she's starting to have some of the longer-term effects, because obviously diabetes then kind of leads into other health issues so to get the diagnosis was quite distressing.” P12*

*Difficulties with sleep*

*“I have enough trouble sleeping, I’m either hyperalert and get up too early, or I can’t get up” P7*

*“I’m woken by pain and occasional flashbacks” P7*

*“I am in pain every day. I don't remember the last time I slept well or slept through the night and didn't wake up in pain like during the night, I don't remember the last time I woke up and wasn't in pain upon waking up.” P12*

*“Doctors did give me zopiclone to help me sleep, make sure I get a good full night’s sleep and stuff because that helps.” P10*

*Cognitive difficulties*

*“I can stupidly start to make things, and I totally forget that I've turned things on so I can like put a coffee machine on and then it's making loads of noise in the background and I'm totally forgot that it's on and I've gone off on something else, and then I'll come back and it's just sort of flowing on the floor.” P5*

*“the mental faculty of being able to remember things, being able to be on the ball. It's like when you have something that will trigger either, like an anxiety attack or panic attack, it's just like your brain seizes up and you can't think at all.” P7*

*“There was an [mental health] appointment I had and, well, I did go but I went at the wrong time. I thought it was later on in the day and it was earlier in the day, so they just discharged me from the service.” P2*

*“But when I found out, obviously with my pain, I didn't know I had mental health until literally say a year and about 1/2 a year later because I was so focused on what was going on with my body.” P3*

*Impact on relationships*

*“I'll get irritable and frustrated. And that causes arguments.” P11*

*“I don't even feel like picking up the phone physically, I mean, even to type because that my fingers hurt me when I’m typing stuff.” P1*

*“romantic relationships, I think has been affected more than normal friendships, and especially from complex PTSD, you know, opening up with someone and sexually being with someone, that's been, you know, different, and I used to not want to be romantic with anyone because of my bowel issues as well” P6*

*“The pain has affected my relationship with [partner], I feel, because I feel like, how does he cope with it and the house? I mean, he's saying, "Sit down and I'll do the dishes".” P9*

*“Even though my kids remind me, every night and every morning they remind me I'll go, ‘Yeah, yeah, I'm going to do it [take epilepsy medication] now’ I still forget, and I feel like a terrible mum.” P10*

*“I don't like to bother people. So they've all got their families, their life. I wouldn't say I don't want to be a burden, just I understand people got stuff going on, so I'll wait my turn.” P11*

*“I won't want to go and socialise or things like that.” P6*

*“I can get triggered so quickly, and it's too much now. I'm not very sociable these days” P8*

*“[Discussing impact of epilepsy] I lost a lot of friends, I lost a lot of people, they all sort of deserted me because I couldn’t drive them places, I couldn’t do anything no more, I couldn’t… yeah, it hurt, it hurt really bad” P10*

*“I go through periods where I cognitively can't reach out to communicate through texts”…“the tank is empty and even just texting someone back and knowing that they're going to text me back, and then we're going to be having a conversation, I'm like, I don't have enough in me to give that.” P12*

*“I don’t have anyone to go and help my son, you know, to look after my son or anything” P3*

Theme 3. Mind-Body Link “when my mind hurts, my body hurts”.

*Mental and physical health affect each other "it's just like one big vicious circle"*

*“Yeah, it [pain] puts be in a bad mood.” P11*

*“someone who’s been through that trauma, I think that there is an association of if you’re in pain, you’re in danger, so you just kind of shut it down so that you can be present and alert.” P12*

*“I’ve come face to face with him [abuser] and I had a complete breakdown. I think I went home and I collapsed. Apparently, I don't remember anything, apparently, I collapsed. I was rushed into [physical health] hospital and pumped full of drugs.” P4*

*“last night, I had quite a bad nightmare, and I woke up and I was, my bed was drenched in sweat, and then I was being in stomach pains.” P6*

*“I was having a panic attack… it was like he was there again doing it to me [flashback], and like I was screaming, crying, and then that would trigger me into it [seizure]. It was like, I'll wake up screaming and I can't breathe and my chest's tight, and it will send me into a panic which will then send me into a seizure.” P10“Going through everything that I've gone through and then having that illness on top of that, it's like, whoa, come on”. P10*

*Difficulties managing co-occurring conditions*

*“I would literally not go. I did not want to leave the house; my house was safe; I just didn't want to go [to physical health appointments].” P4*

*“I was given anaesthetic as well, and one of the traumas, when I was 15, I was drunk, so having that and going into the colonoscopy like, well, it makes you feel very vulnerable… because all the colonoscopy people were all men, which, you know, I can't face that, but the first time I had it, I just remember he [Doctor] was smiling, I just felt really scared.” P6*

*“I'm not gonna let it stop me going to the meeting [peer support group] on Wednesday, there's no way, like, I'll keep going there, I'll take painkillers, but I'll go there.” P9*

*“I couldn’t speak in my therapy session [due to physical health]. We had to not do that session” P4*

*“they say to improve your head, your mental health, go out and exercise, go for a walk. But I can't [due to pain], damned if I do, damned if I don't.” P11*

*Service-users’ variable insights into a mind-body link*

*“I think, in that way that’s linked, and I think my mental health affects the way my bowel and abdominal, like, body works” P6*

*Interviewer: “do you see a link between your mental and physical health?” Participant: “they're two separate things, yeah, it's two separate things for me.”…“when I'm in pain I go into the bedroom and I'm lying down and I just keep thinking, I'm not in [custody], I'm not in [custody], I'm back on my own bed now” Interviewer: “So when you're in pain you end up lying in your bed more, and that's when the thoughts about the trauma [custody] come?” Participant: “Yeah.” P9*

*“I'm learning more from speaking to you from the point of view that maybe what I'm experiencing is interlinked. But it's because you always think, it's just you having a bad day and thinking well, you know, but when I, when you speak, it makes sense that what I'm feeling is actually or could be linked.” P5*

*Interviewer: “Do you think there's a link between your physical and mental health?” Participants “No…Now I don't. If you would have asked me that a few years ago, I probably would have said yes *laughs* Because…what's changed is, like I said, I've been heard, you know, I've been through tests and there is actually something wrong. It's not something that I felt that I was being questioned about. You know, I wasn't having to double think myself and think in my head like, is it really real, you know?” P3*

Theme 4. Negative Core Beliefs About Self, Others and Health "I feel like a broken biscuit that no one wants"

*Feeling out of control with health "I can't control it. It controls you.*

*"“I was feeling crap that I couldn't manage my body, and it gets, like, a cycle. I have no control over my body.”… “I wanted to be better or have a bit more control before I was around other people”…”I didn’t like anyone seeing me vulnerable.” P4*

*“I feel that's [abdominal pain] completely uncontrollable.” P6*

*“I wish I had something to help me to control that [cPTSD], to calm me down, I know the stones and that [grounding technique], but when that stuff doesn’t work, I wish there was something to help to control me so that I’m not in that mood, I’m not in that scary place.” P10*

*“I have no idea. No idea, no control over that [cPTSD] at all.” P1*

*Interviewer: “Do you see it changing in the future?” Participant “I hope so. Please, God, I hope so. Otherwise, I don't know what I'm gonna do, because I can't keep on like this,” P9*

*The self as not good enough, unlovable and worthless*

*“They’re probably thinking, ‘what’s the matter with him? What’s going on? That is [FND] a load of rubbish’ you know.” P5*

*“They see me as a ‘strong, confident out there’ personality, because that's the persona I put on because I don't want anyone to see the weak, the real me, because when I was that weak real me, I was taken advantage of too many times.” P10*

*“You go through phases of, when there is traumas and you don't know what it is, I think am I making this up? Am I a layabout, and I just don't want to admit it?” P8*

*“It's not fair that it affects me like that because then I can't get my life back, you know, it's not. I can't, I can't get my life back. I can't be normal again.” P10*

Frequency of Codes

| Theme | | Number of Codes | Number of Participants |
| --- | --- | --- | --- |
| Mind-body link “when my mind hurts, my body hurts” | | 135 | 12 |
| Difficulties managing co-occurring conditions | 37 | 11 |  |
| Mental and physical health affect each other “it’s just like one big vicious cycle” | 5 | 12 |  |
| Service-users’ variable insights into mind-body link | 31 | 12 |  |
| Negative core beliefs about self, others and health "I feel like a broken biscuit that no one wants" | | 142 | 12 |
| Feeling out of control with health "I can't control it. It controls you." | 65 | 11 |  |
| The self as not good enough, unlovable and worthless | 56 | 11 |  |
| Beliefs of high danger and vulnerability | 21 | 7 |  |
| Negative impact of conditions on well-being | | 218 | 12 |
| Impact on relationships | 51 | 11 |  |
| Cognitive difficulties | 26 | 11 |  |
| Difficulties with sleep | 7 | 6 |  |
| Emotional toll of living with conditions | 67 | 11 |  |
| Reduced meaningful activity | 67 | 12 |  |
| Coping behaviours having a negative impact on health | | 136 | 12 |
| Substance use | 41 | 10 |  |
| Poor self-care | 5 | 3 |  |
| Avoidance | 79 | 12 |  |
| Lack of exercise | 11 | 7 |  |
